# Supplementary material for: A Theoretical Justification for Single Molecule Peptide Sequencing
Source: PLoS Comput Biol. 2015 Feb 25;11(2):e1004080. doi: 10.1371/journal.pcbi.1004080 (PMC4341059; doi:10.1371/journal.pcbi.1004080)
Supplement: S1 Text — (PDF) [file pcbi.1004080.s001.pdf]

Text S1: Detailed pseudo-code describing the algorithm employed for the simulation

Definitions and Input of experimental parameters:

proteome is the set of all **protein** species. Each **protein** is a sequence of amino acids represented as a sequence of **tuples (aa_i, s_i)** where **aa_i** is the amino acid at position **s_i**. The **tuples** are sequenced and positions are indexed from the N- to the C- terminuses of the protein, with the first amino acid having position **1**.

Amino acid **cleave** indicating site at which protease is active. Proteolysis takes place at the carboxyl side of the amino acid. Example: For cyanogen bromide, **cleave** = Met.

Mapping **labels** from set of amino acids to dyes used to label them
Example: **labels** = {Lys: red, Tyr: green} indicates lysines are labeled using a red dye and tyrosines are labeled with a green dye

Amino acid **attachment** indicating which amino acid is used to functionalize peptides to the slide
Example: **attachment** = Cys indicates peptides are functionalized via cystines

Probability **u** ϵ [0, 1] of unsuccessfully labeling an amino acid. This occurs when an amino acid intended to be labeled per **labels** fails to covalently bond to its dye, or the dye that bonds is defective before the experiment begins. **u** is constant across all **labels**.

Probability **p** ϵ [0, 1] of the Edman cycle successfully cleaving off the N-terminal amino acid from a peptide.

Photobleaching constant **b** ϵ [0, ∞) indicating the photobleaching half-life of all fluors.

Number of experimental **cycles** the sample will be subjected to.

Function **random()** is provided by the system and yields random floating point numbers in [0, 1].

Function **binomial(x, y)** is provided by the system and returns the binomial coefficient $\binom{x}{y}$

e is Euler's constant.

Function **sort()** sorts **tuples (aa_i, s_i)** in by s_i in ascending order

Each protein is sampled a **simulation_depth** number of times.

Algorithm section 1: Definition of prefix trie used to collate simulation results and associated utility functions

Definitions:

A **node** in the trie stores three items:

1. **tuple (aa_i, s_i)**
2. references to all **children nodes** by their **tuples (aa_i, s_i)**; for simplicity, we omit the creation of child nodes in this pseudocode and assume they all exist
3. **counters** for all proteins, *i.e.* a mapping from the **proteome** to the set of integers, notated by **counter[protein]**; all **counters** are initialized to 0

The **root node** stores only references to all **children nodes**

Each sequence of **tuples (aa_i, s_i)** uniquely maps to a node in the trie by walking the trie starting from the root node, with each successive **tuple (aa_i, s_i)** indicating the child node to visit next. The sequence is mapped to the last node the walk arrives at. See function **increment_counter** below for an illustration.

Functions:

```
FUNCTION increment_counter(sequence of tuples (aa_i, s_i), protein):
    current_node ← root node
    FOR tuple (aa_i, s_i) IN sequence of tuples:
        current_node ← child (aa_i, s_i) of current node
    #current_node is now the node that the sequence of tuples maps uniquely onto
    counter[protein] ← counter[protein] + 1
FUNCTION recursive_traverse(node):
    list_of_nodes ← (node) #list of all child nodes including self
    FOR node IN children nodes:
        list_of_nodes ← list_of_nodes + recursive_traverse(node)
    RETURN list_of_nodes
```

Algorithm section 2: Experiment initialization

```
peptides[protein] = NULL
  #this will store all peptides proteolysed from protein that are hybridized to the
  #surface

FOR protein IN proteome:
    peptides ← proteolyze protein using cleave
  #peptides is the set of all subsequences of the protein
  #partitioned after tuples with aa_i=cleave; for example,
  #((K, 1) (M, 2)(C, 3)(M,4)) would yield the set
  #{  ((K, 1), (M,2)),   ((C, 3), (M, 4))  }
    FOR peptide IN peptides:
        IF attachment NOT IN peptide:
            discard peptide #peptides not having attachment cannot attach to the surface and are
                            #washed away
    FOR peptide IN peptides:
        FOR tuple (aa_i, s_i) IN peptide:
            IF aa_i NOT IN labels:
                discard tuple from peptide #ignore unlabeled amino acids
    peptides[protein] ← peptides
```

Algorithm section 3: Monte Carlo simulation

```
FUNCTION simulate(peptide, protein):
#the sequence of tuples in peptide is copied for every call of this function and is manipulated below
    sequence ← copy(peptide)

    ###simulate fluor label failure
    FOR tuple (aa_i, s_i) IN sequence:
        IF random() < u:
            discard (aa_i, s_i) from the sequence
    ###end of fluor label failure section

    ###simulate Edman failure
```

```
cumulative_delay = 0 #temporary variable keeping track of total Edman failures
FOR tuple (aa_i, s_i) IN sequence:
        d ← s_i IF this is the first tuple in the sequence ELSE s_i – s_{i – 1}
          #distance between consecutive labels
        delay_sample = random() #generate random point for delay probability distribution
        delay = 0 #keep track of delays for interval between (aa_i, s_i) and (aa_{i – 1}, s_{i – 1})
        accumulator = 0 #temporary variable for accumulating delay probabilities
        #map delay onto [0, 1] via its probability distribution
        WHILE:
                binomial_pdf = 0 #binomial probability density function
                IF random_delay = 0:
                        binomial_pdf ← p^d
                ELSE:
                        binomial_pdf ← binomial(d – 1, d – 1 + delay) * p^d *(1 - p)^delay  -
                                                binomial(d – 1, d – 2 + delay) * p^d *(1 - p)^(delay - 1)
                accumulator ← accumulator + binomial_pdf
                #test if this was the delay chosen by delay_sample
                IF accumulator ≥ delay_sample:
                        BREAK
                ELSE:
                        delay ← delay + 1
        cumulative_delay ← cumulative_delay + delay
        (aa_i, s_i) ← (aa_i, s_i + cumulative_delay)
          #delay aa_i in fluorosequence due to all prior Edman failures
 #simulation assumes Edman cannot proceed past the first amino acid hybridized to the surface
        IF aa_i = attachment:
 #although Edman cannot reach them, the delay still affects fluors after attachment due to
 #photobleaching
                FOR (aa_j, s_j) IN sequence:
                        IF j > i:
                                (aa_j, s_j) ← (aa_j, s_j + cumulative_delay)
                BREAK
###end of Edman failure section

###simulate photobleaching
#first loop photobleaches fluors before the first attachment, because
# Edman cannot proceed past it
#second loop (further below) photobleaches fluors after first attachment
FOR (aa_i, s_i) IN sequence:
#this IF statement stops the first loop at the first attachment
        IF aa_i = attachment:
                BREAK
        photobleach_sample = random()
          #random point for photobleaching probability distribution
        accumulator = 0 #temporary variable for accumulating photobleaching probabilities
        exposures = cycles + 1 IF cycles < s_i ELSE s_i #number of exposures for the fluor
        FOR k FROM 0 TO exposures - 1:
                accumulator ← accumulator + e^(-bk)
                IF accumulator * (1 – e^(-b)) ≥ photobleach_sample:
                        (aa_i, s_i)  ←  (aa_i, k + 1)
                        BREAK
```

```
#second loop photobleaches fluors after first attachment
FOR (aa_i, s_i) IN sequence:
    #this IF statement ignores all fluors before the first attachment
    IF aa_i = attachment:
        CONTINUE
    photobleach_sample = random()
      #random point for photobleaching probability distribution
    accumulator = 0 #temporary variable for accumulating photobleaching probabilities
    exposures = cycles #number of exposures for these fluor is always all cycles
    FOR k FROM 0 TO exposures - 1:
        accumulator ← accumulator + e^-bk
        IF accumulator * (1 – e^-b) ≥ photobleach_sample:
            (aa_i, s_i)  ←  (aa_i, k + 1)
            BREAK
###end of photobleaching section

#sort sequence by final observations and collate result into trie
sequence ← sort(sequence)
increment_counter(sequence, protein)

#main simulation loop
FOR protein IN proteome:
    FOR k FROM 0 to simulation_depth:
        FOR peptide IN peptides[protein]:
            simulate(peptide, protein)
```

Algorithm section 4: Count identified proteins

```
identified_proteins = {} #set of all proteins considered classified

FOR node in recursive_traverse(root node):
    total_source_proteins = 0 #calculate total number of times the fluorosequence mapping to this node
                              #has been observed
    FOR protein IN counters:
        total_source_proteins ← total_source_proteins + counters[protein]
    FOR protein IN counters:
        IF counters[protein] > 10 AND counters[protein] / total_source_proteins > 0.90:
          identified_proteins ← identified_proteins + protein

RETURN identified_proteins
```
